# Supplementary material for: Conditional cooperation in group contests
Source: PLoS One. 2020 Dec 23;15(12):e0244152. doi: 10.1371/journal.pone.0244152 (PMC7757887; doi:10.1371/journal.pone.0244152)
Supplement: S1 Appendix — (PDF) [file pone.0244152.s001.pdf]

S1 Appendix. Supplementary material - Robustness check

	Decrease contribution	No change in t	Increase contribution	# observations
Contribution < 80% Average in t-1				
overall	21.07%	30.37%	48.55%	484
winner / loser	16.88% / 25.10%	37.55% / 23.48%	45.57% / 51.42%	237 / 247
deserved winner / loser	18.60% / 24.32%	37.21% / 24.32%	44.19% / 51.35%	129 / 148
chance winner / loser	14.81% / 26.26%	37.96% / 22.22%	47.22% / 51.52%	108 / 99
Contribution = Average in t-1 +/- 20%				
overall	40.20%	24.51%	35.29%	204
winner / loser	39.05% / 41.41%	29.52% / 19.19%	31.43% / 39.39%	105 /99
deserved winner / loser	41.67% / 21.28%	31.67% / 12.77%	26.67 % / 65.96%	60 / 47
chance winner / loser	35.56% / 59.62%	26.67% / 25.00%	37.50% / 15.38%	45 / 52
Contribution > 120% Average in t-1				
overall	44.68%	19.68%	35.64%	376
winner / loser	41.58% / 47.85%	25.26% / 13.98%	33.16% / 38.17%	190 / 186
deserved winner / loser	40.54% / 37.14%	29.73% / 7.62%	29.73% / 55.24%	111 / 105
chance winner / loser	43.04% / 61.73%	18.99% / 22.22%	37.97% / 16.05%	79 / 81

S1 Table. Participants' reaction to if their contribution was 20% less than the group average / group average +/- 20% / 20% more than the group average in the previous round
