# Supplementary material for: Conditional cooperation in group contests
Source: PLoS One. 2020 Dec 23;15(12):e0244152. doi: 10.1371/journal.pone.0244152 (PMC7757887; doi:10.1371/journal.pone.0244152)
Supplement: S2 Appendix — (PDF) [file pone.0244152.s002.pdf]

S2 Appendix. Supplementary material - Downward trend in contribution In S1 Fig we present the downward trend in contributions. Average contribution in round 1 is the only observation that does not fit into the general trend.

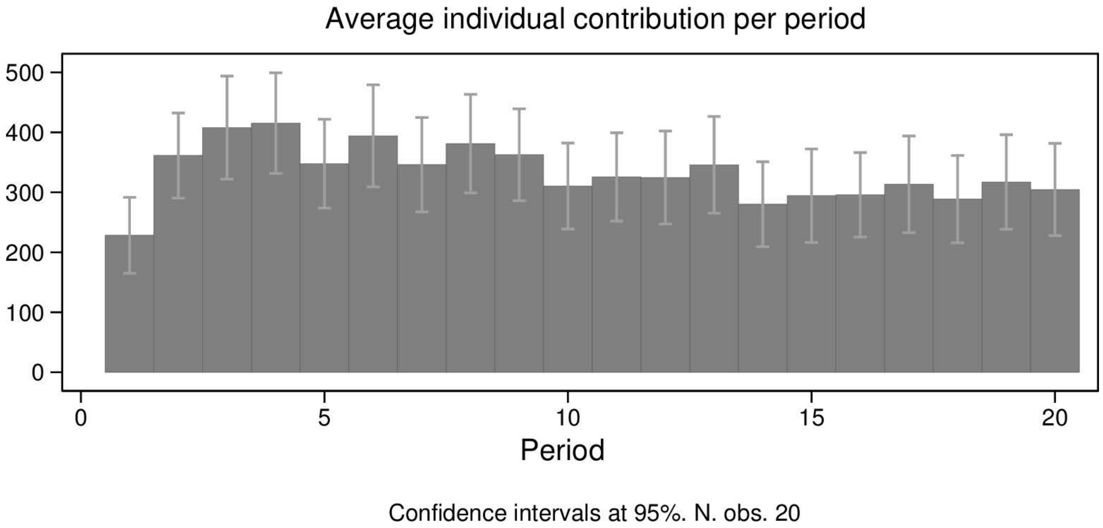

S1 Fig. Average individual contribution per round

In the first five rounds, the average contribution was equal to 353.3; for rounds 6 to 10 it was 371; for rounds 11 to 15 319.1, while in the last five rounds it equaled 303.8. We see a downward trend. We look for the presence of a downward trend via a within-subject analysis by comparing decisions in the first and last five rounds. A statistically significant lower effort at 10 % in the last 5 rounds is found relative to the first 5 rounds (Wilcoxon signed-rank test, p-value= 0.085).
