# Supplementary material for: Conditional cooperation in group contests
Source: PLoS One. 2020 Dec 23;15(12):e0244152. doi: 10.1371/journal.pone.0244152 (PMC7757887; doi:10.1371/journal.pone.0244152)
Supplement: S3 Appendix — (PDF) [file pone.0244152.s003.pdf]

**S3 Appendix.** S2 Table is the analogue of Table 4, but we replace the standard errors with 95% confidence intervals.

| Dependent variable: Percentage change in individual contribution in round $t$ with respect to $t$-$1$ | | | | |
|---|---|---|---|---|
| | Spec. 1 | Spec. 2 | Spec. 3 | Spec. 4 |
| i's contribution (*t-1*) w.r.t avg group contribution (*t-1*) in pc | -0.081*** | -0.074*** | 0.001 | -0.060 |
| | [-0.123,-0.040] | [-0.115,-0.032] | [-0.073,0.074] | [-0.136,0.016] |
| Round | -0.609*** | -0.628*** | -0.600*** | -0.622*** |
| | [-1.045,-0.173] | [-1.061,-0.194] | [-1.033,-0.167] | [-1.050,-0.193] |
| Winner by chance (*t-1*) | | -2.993 | -3.310 | -2.780 |
| | | [-9.765,3.778] | [-10.07,3.447] | [-9.498,3.939] |
| Winner deserved (*t-1*) | | -4.074 | -5.301 | -5.388 |
| | | [-10.43,2.283] | [-11.72,1.116] | [-11.96,1.184] |
| Loser by chance (*t-1*) | | -13.375*** | -14.593*** | -14.447*** |
| | | [-20.18,-6.572] | [-21.45,-7.737] | [-21.41,-7.480] |
| i's wrt gr's contribution (*t-1*) x winner chance (*t-1*) | | | -0.061 | -0.056 |
| | | | [-0.138,0.015] | [-0.133,0.021] |
| i's wrt gr's contribution (*t-1*) x winner deserv (*t-1*) | | | -0.057 | -0.044 |
| | | | [-0.143,0.029] | [-0.131,0.042] |
| i's wrt gr's contribution (*t-1*) x loser chance (*t-1*) | | | -0.136*** | -0.109** |
| | | | [-0.227,-0.045] | [-0.200,-0.017] |
| Covariates | | | | YES |
| Constant | 1.147 | 6.187* | 7.674** | -67.432 |
| | [-4.227,6.521] | [-0.459,12.83] | [0.936,14.41] | [-157.5,22.68] |
| $R^2$ | 0.020 | 0.034 | 0.043 | 0.079 |
| Observations | 1064 | 1064 | 1064 | 1064 |

Standard errors in parentheses.
Random effects linear panel regression model.
Dependent variable normalized.
$^{*}$ $p < 0.10$, $^{**}$ $p < 0.05$, $^{***}$ $p < 0.01$

**S2 Table.** Determinants of the percentage change in individual's contribution in $t$ with respect to *t-1*. Random effects linear panel regression model.
