# Supplementary material for: Conditional cooperation in group contests
Source: PLoS One. 2020 Dec 23;15(12):e0244152. doi: 10.1371/journal.pone.0244152 (PMC7757887; doi:10.1371/journal.pone.0244152)
Supplement: S4 Appendix — (PDF) [file pone.0244152.s004.pdf]

**S4 Appendix. Supplementary material - Instructions**

The language of the instruction was Spanish as we ran the experiment in Spain. Here we present the English version of the instructions. The instructions clarified the different tasks related to the Group Contest (Phase 1), Social Value Orientation (Phase 2), Public Goods game (Phase 3), Bomb Risk elicitation Task (Phase 4) and Competitiveness (Phase 5), as well as the Questionnaire.

## Welcome to the experiment

Welcome and thanks for participating in this experiment! Please, read carefully these instructions. The instructions are the same for all participants with whom you are going to interact during the experiment.

This is an experiment to study how individuals make decisions. We are interested in what individuals do on average.

Do not think that we expect any particular behavior from you. However, keep in mind that your behavior affects the amount of money that you may earn.

Next, you will see a series of instructions explaining how the experiment works and how you can use the computer during the experiment.

Please, do not either speak to or disturb the other participants during the experiment. If you need help, raise your hand and wait quietly. You will be attended as soon as possible. From now on, no type of communication is allowed with other participants. Please, switch off your mobile phone. If you do not comply with the rules, you will be dismissed and you will not receive any compensation for your participation.

The structure of the experiment is the following:

- PHASE 1
- PHASE 2
- PHASE 3
- PHASE 4
- PHASE 5
- QUESTIONNAIRE

You will have to make decisions in all phases as we will explain during the experiment. All your decisions will be treated confidentially.

The final payoffs will be the sum of two payoffs. The first payoff is the one that you obtain in Phase 1 and the second payoff is the one that is picked randomly from the payoffs related to the rest of the phases.

We record the earnings during the experiment as tokens that will be converted into euros at an exchange rate that we specify in each phase. In all cases, more tokens imply more euros.

After finishing the 5 phases, you will be asked to fill in a questionnaire and then we will pay your earnings in euros in private.

Read carefully the following instructions in order to know what are the decisions that you are going to make and how you can earn money.

In this phase of the experiment you will be a member of a group composed of 4 persons in the room. These 4-person groups will be formed randomly by the computer. You will not know the identities of the persons who are in your group, neither will they know your identity. Hence, identity of all members of the groups will remain anonymous. From the 56 individuals in the room, 14 4-person groups will be formed.

This phase consists of 20 rounds.

Throughout this phase, your group will play against another group, so your group and the other group will form a pair of rival groups. You will be member of the same group during the 20 rounds and you will play against the same rival group in each of the 20 rounds. The game consists in that the rival groups compete for a prize, as we will explain it in detail now.

At the beginning of each round you will receive an individual endowment of 1000 tokens. You can use this individual endowment of 1000 tokens to buy 'competition tokens' for the public account of your 4-person group. Each competition token costs 1 token of your individual endowment, hence you may buy at most 1000 competition tokens. The tokens of your individual endowment that you do not spend on buying competition tokens for the public account, will remain on your individual account. Similarly, the other 3 members of your group will have an endowment of 1000 tokens that they can use to buy competition tokens for the public account of your 4-person group.

At the end of each round, when each individual in the room has chosen how many competition tokens to buy, a random process similar to a wheel of fortune determines which group (the group that you belong to or the rival group with whom your group competes) wins the prize. The prize is 4000 tokens. The probability of winning the prize depends on the amount of competition tokens that your group has and on the amount of competition tokens acquired by the rival group. More concretely, the following happens.

The 'wheel of fortune' will be divided in two parts with different colors. One part of the wheel belongs to your group and the other part to the rival group. The size of the parts of the wheel represent exactly in a proportional way the amount of competition tokens acquired by your group and the rival group. For example, if your group and the rival group have acquired the same amount of tokens, then each group has 50% of the 'wheel of fortune'. If your group has acquired twice as many tokens as the rival group, then your group has two thirds of the wheel and the rival group has the remaining one third. Once the division of the wheel is determined by the competition tokens of the groups, the wheel starts to spin and stops randomly after a while. The wheel has an indicator in the position of 12 hours of a clock. The prize will be won by the group above whose color the indicator of the wheel is. Imagine that your group has acquired twice as many competition tokens than the other group and the color of your group is red, while the color of the rival group is blue. In this case, two thirds of the wheel will be red and one third blue. If after stopping the wheel the indicator is above a part of the wheel that is red, then your group wins the prize. However, if the indicator is above the blue part, then the prize goes to the rival group.

Therefore, the probability that your group wins the prize increases in the amount of competition tokens acquired by your group. In the same vein, the more tokens the rival group acquires, the higher is the probability that the rival group wins the prize. If one of the group does not acquire competition tokens, while the other group acquires tokens, then the group with the tokens wins with certainty. If none of the groups acquires tokens, the prize will be assigned randomly to one of the groups.

If your group wins the prize, then the 4000 tokens will be divided equally among the members of the group, independently of how many competition tokens each member of

the group bought. That is, if a group wins the prize, the members of the group will receive 1000 tokens. In this case, the total amount of tokens obtained in the round would be the tokens of your initial endowment not used to buy competition tokens and your share of the prize, 1000 tokens.

If your group does not win the contest, you will not receive anything from the prize. In this case, your payoff in the round would be the initial endowment minus the tokens used to buy competition tokens.

Imagine that you use 350 tokens from your initial endowment of 1000 tokens to buy competition tokens. Suppose that your group wins the contest and obtains the prize. In this case, your payoff is (1000-350)+1000=1650 tokens in that round. Now assume that the other group wins the contest. Then your payoff in that round would be 1000-350=650 tokens. The numbers used in this example are fictitious.

The earnings will depend always on the amount of competition tokens that the members of the groups buy and on the result of spinning the wheel of fortune.

At the end of each round, after determining the winner of the contest, the earnings of the round will be computed.

Moreover, at the end of each round you will receive the following information:

- your contribution to the common cause (that is, how many competition tokens you have bought);
- the total contribution of your group (that is, the total number of competition tokens bought by the members of your group) ;
- the total contribution of the rival group (that is, the total number of competition tokens bought by the members of the rival group);
- if your group has won the contest or not;
- your individual earning in the given round.

Your final earning in this phase of the experiment will be the sum of the earnings obtained in 5 randomly chosen rounds from the 20 rounds that you play in this phase.

**The exchange rate in this phase of the experiment is the following:**

1000 tokens = 1.2 Euros (that is, 1 token = 0.12 Euro cents).

For example, if you earn 5000 tokens then you will receive 6 Euros.

**Phase 2**

Read carefully the following instructions in order to know what are the decisions that you are going to make and how you can earn money.

In this phase of the experiment you will be paired with another person in the room, that is, you will be part of a two-person group. The two-person groups are formed randomly by the computer. You will not know the identity of the other person who will be in your group, neither will she / he know that you are the other member of the group. Hence, anonymity is maintained. The decisions that any individual of this room makes are anonymous as well.

This phase consists of 6 experimental rounds.

In each round you will see on the screen a sequence of 9 pairs of numbers. In each pair of numbers, one of the numbers is your payoff and the other number is the payoff of the other person of your group. Your task in this phase is to choose the pair of numbers that you prefer in each round.

Once everybody in the room has decided in each of the six rounds, we will proceed with the calculation of the payoffs of this phase of the experiment.

First, the computer chooses randomly one of the 6 rounds that we will use for the payoffs.

Once the payoff-relevant round is selected, the computer will pick randomly one of the persons in each group who will be the Elector.

There is a 50% chance that you will be the Elector and and a 50% chance that the other person in your group will be the Elector.

In this phase, the payoff of each person in the room will be the decision made by the Elector in the group in the round that has been selected for payoff.

**The exchange rate in this phase of the experiment is the following:**
1 token = 0.02 Euros (that is, 1 token = 2 Euro cents).
For example, if you earn 100 tokens, then you will receive 2 Euros.

**Phase 3**

Read carefully the following instructions in order to know what are the decisions that you are going to make and how you can earn money.

In this phase of the experiment you will be part of a 4-person group. The four-person groups are formed randomly by the computer. You will not know the identity of the other persons who will be in your group, neither will they know that you are a member of the group. Hence, anonymity is maintained. The decisions that any individual of this room makes when interacting with the other members of the her / his group are anonymous as well.

This phase consists of 1 experimental round.

At the beginning of the round everybody in the room will receive an endowment of 1000 tokens. Your task is to decide how much of your endowment to assign to the common account of the group. That is, you have to decide how many tokens of the 1000 that you have you want to contribute to an account that you share with the other members of your group. The other members of your group will make the same decision, that is how many tokens from the initial endowment they assign to the common account of your group.

Once everybody in the room has decided, we will proceed with the calculation of the payoffs of this phase of the experiment.

In this phase, the individual payoff of each member of the group depends on her / his decision, on the decisions of the other members of the group and on a multiplier. We will explain the payoff in detail:
Your earning = initial endowment - contribution to the common account + 0.4* (common account of the group)
$P_i = 1000 - x_i + 0.4(x_1 + x_2 + x_3 + x_4)$, where

- $P_i$ is the individual earning
- 1000 is the initial endowment
- $x_i$ is your contribution to the common account
- 0.4 is the multiplier
- $x_1 + x_2 + x_3 + x_4$ is the sum of the contributions of each member of the group, that is the total of tokens accumulated in the common account of your group

For example, if you contribute 250 tokens to the common account, while the total contribution is 800, then you earn (1000-250)+0.4*800=1070 tokens.

**The exchange rate in this phase of the experiment is the following:**

1 token = 0.002 Euros (that is, 1 token = 0.2 Euro cents).

For example, if you earn 500 tokens, then you will receive 1 Euro.

**Phase 4**

Read carefully the following instructions in order to know what are the decisions that you are going to make and how you can earn money.

In a store there are 100 boxes, numbered from 1 to 100. In one of the boxes there is a bomb. In the other 99 boxes there is money (each of them contains money). You do not know which box contains the bomb, but you know that it could be in any of the boxes with the same probability.

Your task in this phase is to choose how many boxes wou would take out from the store. The boxes are numbered and will be taken out in numerical order (starting with box 1). That is, if you want to take out 20 boxes, then the boxes numbered from 1 to 20 will be collected. If you want to take out 57 boxes, then the boxes from 1 to 57 will be collected.

Once everybody in the room has decided, we will proceed with the calculation of the payoffs of this phase of the experiment.

At the end of this phase, the computer will choose randomly a number between 1 and 100 to determine in which box the bomb is. If the bomb is in one of the boxes that you collected from the store, then you will earn nothing in this phase of the experiment. However, if the bomb is not in the boxes that you took out of the store, then you may open the boxes and you will receive a token for each box.

Next, we will show some examples to illustrate how you can earn money in this phase:

Case A) Imagine that you decide to collect 7 boxes and the bomb is in the box 42. Since you did not collect the box with the bomb, you earn a token for each of the 7 boxes that you collected, that is your earnings will be 7 x 1 token = 7 tokens.

Case B) Imagine that you decide to collect 35 boxes and the bomb is in the box 42. Since you did not collect the box with the bomb, you earn a token for each of the 35 boxes that you collected, that is your earnings will be 35 x 1 token = 35 tokens.

Case C) Imagine that you decide to collect 52 boxes and the bomb is in the box 42. Since you did collect the box with the bomb, you earn zero token in this phase.

Case D) Imagine that you decide to collect 68 boxes and the bomb is in the box 73. Since you did not collect the box with the bomb, you earn a token for each of the 68 boxes that you collected, that is your earnings will be 68 x 1 token = 68 tokens.

Case E) Imagine that you decide to collect 10 boxes and the bomb is in the box 7. Since you did collect the box with the bomb, you earn zero token in this phase.

**The exchange rate in this phase of the experiment is the following:**

1 token = 0.1 Euros (that is, 1 token = 10 Euro cents).

For example, if you earn 50 tokens, then you will receive 5 Euro.

**Phase 5**

Read carefully the following instructions in order to know what are the decisions that you are going to make and how you can earn money.

In this phase you will have to complete 4 tasks. None of these tasks will take more than 5 minutes. At the end of this phase, the computer will choose randomly one of the 4 tasks and you will receive your earnings based on your performance in that task. The computation of the earnings varies between tasks as we will inform you before starting each of the tasks.

*Task 1*

In Task 1, you will see on the screen a series of sliders that you have to work with during a minute. Each slider can be moved along the integer numbers going from 0 to 100. At the beginning of the task each slider is positioned at 0. Your task consists in moving as many sliders as you can in a minute to the number 50 with the help of the mouse. On the right hand side of each slider you will see the number at which the slider is positioned. You can use the mouse to readjust the position of the sliders as many times as you need.

If Task 1 is chosen for payment in this phase of the experiment, then you will receive 1 token for each slider positioned at the number 50. Your payoff will not decrease in the number of sliders not positioned at the number 50. That is, the payment depends on the number of sliders positioned at the number 50.

**The exchange rate in this phase of the experiment is the following:**
1 token = 0.15 Euros (that is, 1 token = 15 Euro cents).
For example, 20 tokens = 3 Euro.

*Task 2*

In this task you will have 1 minute to work with a set of sliders. This task, as the previous one, consists in positioning sliders at the number 50. However, in this task your earning depends on your performance in relation to the performance of the members of the group that you belong to. That is, your earning depends on the number of sliders positioned at the number 50 and the quantity of sliders that the other members of your group positioned at the number 50. Each group in the room will be formed by 4 individuals, so you will be in a group with 3 other persons in the room. The person of the group that has the highest number of sliders positioned at the number 50 in a minute, will receive 4 tokens for each slider at the number 50, while the other members of the group will receive 0 token.

You will not be informed about your performance in this tournament until the end of this phase of the experiment. If there is a tie, the winner will be chosen randomly by the computer.

Remember that in this task the only one to win tokens and hence money is the one that positions the highest number of sliders correctly at the number 50.

**The exchange rate in this phase of the experiment is the following:**
1 token = 0.15 Euros (that is, 1 token = 15 Euro cents).
For example, 20 tokens = 3 Euro.

*Task 3*

In this task, you will have 1 minute to work with sliders. This task, as the previous ones, consists in positioning the sliders at the number 50. However, at the beginning of this task you will have to decide the way that you want to be paid, that is the way that we compute your earning.

If you choose piece-rate payment, then you will earn 1 token for each slider positioned at the number 50.

If you choose the tournament payment, then your performance in this task will be compared with the performance of the other members of your group in task 2. If the amount of sliders positioned at the number 50 is larger than that of the other members of your group, then you will receive 4 times more tokens per slider than in the piece-rate payment, that is 4 tokens for each slider positioned at the number 50. If in this task the number of sliders positioned at the number 50 is less than that of any of the members in

your group in task 2, then you will earn 0 token. We will not inform you about your performance in the tournament until the end of this phase. In case of a tie, the winner will be chosen randomly by the computer.

If you are the person in your group with the highest number of sliders positioned at the number 50, you will receive 4 tokens for each slider at the number 50, otherwise you will earn 0 token.

**The exchange rate in this phase of the experiment is the following:**
1 token = 0.15 Euros (that is, 1 token = 15 Euro cents).
For example, 20 tokens = 3 Euro.

*Task 4*

In this task you will not have to work with the sliders. Notwithstanding, you can obtain some additional earning for the amount of sliders positioned at the number 50 in task 1. You have to choose the way of payment that you would like to be applied to compute your earnings in task 1. You can choose the piece-rate payment or the tournament payment.

If this task, that is task 4, is chosen for payment in this phase of the experiment, then your earnings will be the following. If you chose the piece-rate payment, then you will earn 1 token for each slider positioned at the number 50 in task 1.

If you chose the tournament payment, then your earnings are determined in relation to the performance of the other members in your group in task 1. Each group in the room will be formed by 4 individuals, so you will be in a group with 3 other persons in the room. If the amount of sliders positioned at the number 50 in task 1 is larger than that of the other members of your group, then you will receive 4 times more tokens than in the piece-rate payment, that is, 4 tokens for each slider positioned at the number 50. If the number of sliders positioned above the number 50 in task 1 is less than that of any of the members of your group in task 1, then you will receive 0 token. Hence, if you choose the tournament payment and you are the one in the group with the highest number of sliders positioned at the number 50, then you will earn 4 tokens for each slider at the number 50, otherwise you will earn 0 token.

In this task, you will see on the screen the amount of sliders that you positioned at the number 50 in task 1 and next you will have to select the way of payment.

**The exchange rate in this phase of the experiment is the following:**
1 token = 0.15 Euros (that is, 1 token = 15 Euro cents).
For example, 20 tokens = 3 Euro.

*Additional questions*

If you answer the next questions correctly, you can earn extra payment. These questions refer to your performance in the previous tasks, compared to those of the other participants.

In task 1, with the piece-rate payment, what do you think your performance was in relation to the other members in your group.

- The best
- The second best
- The third best
- The fourth

In task 2, with the tournament payment, what do you think your performance was in relation to the other members in your group.

- The best
- The second best
- The third best
- The fourth

For each correct answer, you will receive 7 tokens.
1 token = 0.15 Euros (that is, 1 token = 15 Euro cents).

**Questionnaire**

(The questionnaire contained questions about the sociodemographics, linguistic and cognitive abilities , and personality characteristics of the participants. The questionnaire is available upon request.)
