# Supplementary material for: Conditional cooperation in group contests
Source: PLoS One. 2020 Dec 23;15(12):e0244152. doi: 10.1371/journal.pone.0244152 (PMC7757887; doi:10.1371/journal.pone.0244152)
Supplement: Attachment — Submitted filename: PLOSone_Review_final.docx [file pone.0244152.s005.docx]

**Editor**

**Journal Requirements:**

When submitting your revision, we need you to address these additional requirements.

**1.** Please ensure that your manuscript meets PLOS ONE's style requirements, including those for file naming. The PLOS ONE style templates can be found at

<https://journals.plos.org/plosone/s/file?id=wjVg/PLOSOne_formatting_sample_main_body.pdf> and

<https://journals.plos.org/plosone/s/file?id=ba62/PLOSOne_formatting_sample_title_authors_affiliations.pdf>

**2.** We note that you have stated that you will provide repository information for your data at acceptance. Should your manuscript be accepted for publication, we will hold it until you provide the relevant accession numbers or DOIs necessary to access your data. If you wish to make changes to your Data Availability statement, please describe these changes in your cover letter and we will update your Data Availability statement to reflect the information you provide.

**Once accepted, we will upload and make public immediately the data on osf.io where we have created a project named Conditional cooperation in group contests (**[**https://osf.io/q96m7/**](https://osf.io/q96m7/)**).**

**3.** We note you have included a table to which you do not refer in the text of your manuscript. Please ensure that you refer to Table 4 in your text; if accepted, production will need this reference to link the reader to the Table.

**We are grateful for spotting the lack of reference to Table 4 in the original version of the manuscript. After introducing a new table in the new version of the manuscript, Table 4 becomes Table 5. We refer to this table in the last paragraph of section “Findings” as follows (new text in bold):**

**“In order to check the occurrence of conditional cooperation, we test the combination of the relevant coefficients (see Table 5).”**

**Additional Editor Comments:**

The two reviewers have agreed that the paper addresses an interesting problem and has good merit. However, there are still some major issues that need to be addressed. Authors should consider the reviewers' comments and suggestions carefully when preparing the revised version.

**We made an effort to address all the comments raised by the referees. We truly believe that their comments improved the paper considerably.**

**Reviewer #1: Overview and general recommendation**

This manuscript presents a behavioral economics experimental study that investigates whether conditional cooperation is present in group contests, a type of game in which groups have to cooperate in order to increase their chances of winning a prize when competing against a rival group. The authors argue that their findings evidence the existence of condition cooperation in group contests and that such behavior is correlated with the success of the group.

The research questions pursued in this manuscript are relevant and aim to shed light into the nature of human decision-making in groups, whether this decision-making is conditional and which factors affect such conditional behavior. Overall, the manuscript is well written and addresses an important subject, as well as provide interesting conclusions. However, I have some major concerns related to the experimental design and the results, which prevent me from recommending the acceptance of the article until they are addressed. If the authors are able to address my concerns or convince me otherwise, I would be eager to change my assessment and recommend acceptance.

**We are grateful to Reviewer #1 for reading carefully our manuscript and for providing valuable feedback**.

**Major comments**

**Experimental design**

**1**. In this experiment, if I understand correctly (please correct me if I did not), participants may actually obtain an (almost) egalitarian distribution of payoffs between their own group and the rival groups if they do never contribute: if no one contributes, then each participant will keep their initial endowment and there is a 50% of chances that each group will receive the prize, so on average both groups should receive the prize as many times over the course of the experiment. However, **Figure** S2 shows that on average participants contributed about 3/10 of their endowment (please note that this Figure lacks **confidence intervals and sample size information**; this should be added). What do the authors think might be the motivation behind this result? It would be interesting for the manuscript if the authors provided some expected model of behavior for the observed results apart from the hypotheses already indicated.

**Certainly, one of the most striking facts in group contests is the high contribution observed, which also occurs in our experiment. We thank the referee for pointing it out because, certainly, it is important to explain this fact. There is a growing experimental literature that documents this phenomenon called overexpenditure and that has identified some of its determinants. Although it is not the scope of our paper, we now briefly introduce this literature in the second paragraph of the paper, as follows (new text is in bold):**

**“Group contests are pervasive, including rent-seeking and lobbying, innovation 15 tournaments and R&D races or sports competitions. The experimental group contest 16 literature consistently finds that average effort level (though often showing a declining 17 pattern) is significantly higher than the equilibrium prediction, a phenomenon known as 18 overexpenditure, see for instance (Abbink et al., 2010; Bhattacharya, 2016; Brookins et al., 2015; Cason et al., 2012, 2017; Chowdhury et al., 2016; Eisenkopf, 2014; Sheremeta, 2011, among others). Some explanations provided by the literature are pure joy of winning (Cason et al. (2018), Price and Sheremeta (2011), Price and Sheremeta (2015), Sheremeta (2010)), bounded rationality (Chowdhury et al., 2014; Fallucchi et al., 2013; Lim et al., 2014), relative payoff maximization (Mago et al., 2016) and social identity (Chowdhury et al., 2016). Surprisingly, the role of conditional cooperation in group contests has not been analyzed yet. In this paper we approach experimentally this question. Some studies… “**

**In the original Figure 1 found in the Supplementary material, S2 Appendix, we have added confidence intervals and number of observations, as suggested.**

**2**. I could not see anywhere what is the participant **show-up fee** of the experiment, i.e., how much does each participant receive just for showing up. Neither did I see any information about the **ethical advice** of the experiment. I think the authors should comment on that and also upload the data of this experiment so that reviewers can check the validity of the results.

**Indeed, the monetary value of the show-up fee paid to participants in our experiment has not been mentioned throughout the text. We have added it to the last sentence of Section 2, called “The experiment” as follows (new text is in bold):**

**“Overall, the experiment lasted two hours and participants earned 16 euros on average (including the show-up fee of 5 Euro and the payment for the experimental games to elicit participants' characteristics.”**

**Regarding the ethics statement, in section The experiment paragraph 2 contains information on the consent that participants read and sign before starting the experiment. This new part reads as follows:**

**“At the beginning of the experiment the participants received a consent sheet that they read and signed (if they agreed) before starting the experiment. This written consent contained information about the experiment, the confidentiality of the data and the anonymity of the decisions. No minors were involved in the experiment.”**

**Once accepted, we will upload and make public immediately the data on osf.io where we have created a project named Conditional cooperation in group contests (**[**https://osf.io/q96m7/**](https://osf.io/q96m7/)**).**

**Experimental results**

1. In Table 2, the authors show the percentage of participants that increase/decrease their contributions with respect to some conditions. Yet, the magnitude of the change is not shown, nor whether the results are significant. I encourage the authors to provide this information, so that the statistics presented in the manuscript are more transparent, and it is easier to understand the magnitude of the effect presented in the paper.

**We thank the referee for pointing out this issue of magnitudes. It is very natural to ask how are the magnitudes related to conditional cooperation. We did not have it in the previous version because in the literature the idea of conditional cooperation is mainly directional, in the sense that it is identified by the move toward the average. For instance, Fischbacher et al. (2001) or Thöni and Volk (2018) define conditional contributors based on if an individual's contribution increases (at least in a weakly monotonic way) in the other individuals' contribution or using correlation measures between own and others' contribution. We mention this in the new version in section Findings. That is why we focused in the earlier version on the direction of the change in contributions, depending if the subject contributed less / more than the average.**

**However, we agree with the referee that it is a relevant question to see that besides the frequency of moves toward the group average, how is the average size of those moves. Hence, we have included a new Table in the paper, Table 3. In this new Table we reproduce the average contribution variation (and its standard deviation) for each of the cases described previously in Table 2.**

**Although the large standard deviations imply that we cannot talk about statistically significant differences among categories, some regularities are observed. In particular, the magnitude of the change is always higher after losing the contest in the previous round that after winning. We analyse the numbers in the following way:**

**“Table 3 provides information on the average size of the change in contribution joint with the standard deviation in each of the cases. When considering the case of having contributed less than the group average in round t-1, we see that size of change (in absolute value) is markedly higher for those who decreased their contribution. This is true overall and also if we condition on winning or losing and on doing so deservedly or by chance. Hence, while upon falling short of the group average in round t-1 the share of those who increase their contribution in round t is higher (and often by a wide margin) than the share of those who decrease, those in the latter group decrease much more on average than those who increase their contribution. This suggests that even if increasing contribution (the choice in line with the idea of conditional cooperation) is the most frequently observed reaction among those who contributed less than average in round t-1, their impact on overall contribution may be neutralized by participants who decrease their contribution in the same condition due to the fact that in the latter group the size of the change is larger. Turning to those who in round t-1 contributed more than the average, we observe that in this group those who decrease their contribution (in line with conditional cooperation), do so by a larger extent than those who increase their contribution. This happens in all cases, except for chance losers. Hence, for those contributing more than the average in the previous round, it is not just the share of those who decrease their contribution in the next round is higher, but also the size of their reaction than the share and size of those who react in the opposite way.”**

The results in this table appear to indicate that there are 22.59% more participants that increase their contributions in comparison to those that decrease them, when the participant had contributed less than the average in the previous round. Likewise, participants that contributed more than the average tend to reduce their contributions in the next round (although the difference here is much smaller, ~9.48%). This seems to confirm the hypothesis that participants tend to approximate their contributions to the average of the group. This is an interesting result by itself. However, the differences between decreasing/increasing contributions between participants who are in groups that won/lost deservedly/by chance are, in general, quite small and it is difficult to see whether this has an effect. Hence, the main effects (the conditional behavior observed for participants that contribute more than the average) should be emphasized.

**We thank the referee for pointing out the lack of clarity in our discussion of Table 2. We also wanted to stress that these differences represent one of the main results, that suggests the existence of conditional cooperation. Now we include this result in Figure 1, as suggested by the referee in a later point as well as by the other referee, and discuss it separately as follows, emphasizing also the asymmetry in conditional cooperation (the phenomenon being stronger for those who contributed less than the average):**

**“Figure 1 indicates the share of participants who decided to increase or decrease their contribution depending on their behavior with respect to the group in the previous round. It shows that participants increase their contribution more frequently after having contributed less than the group average in the previous round, and that they decrease their contribution more frequently after having contributed more than the group average in the previous round. This suggests that there is conditional contribution in the group contest, the phenomenon being more emphatic upon having contributed less than the group average in the previous round.”**

**In the regression analysis we show which factors prove to be significant.**

2. The authors perform a panel regression with different combinations of parameters (specifications) to check significance. They say “In specifications 1 and 2 the coefficient of the first variable is negative and significant, indicating that having contributed more or less than the average and the magnitude of deviation provokes a strong move in the opposite direction”. I agree with the first part of the phrase, but significance does not indicate the magnitude of the effect, thus I do not see how the authors can backup that there is a strong move in the opposite direction (see [1] and [2]). In fact the coefficient of the regression is -0.081.

**We agree with the referee in that our previous version was not really describing our results. We have eliminated the term “strong” and the reference to the magnitude of the move.**

**“In specifications 1 and 2 the coefficient of the main explanatory variable is negative and significant, indicating that having contributed more (or less) than the average provokes a move in the opposite direction.”**

It would be really helpful to be able to see the **R^2** of the regression to understand whether it is actually providing a good fit for the data. Additionally, some of the effects tested are correlated: information about winning/losing the game and about the contributions is shown at the same time to the participants, thus, its effect on their actions in the next round might be correlated. This can hinder the trust in the p-values results.

**We agree with the referee that we should have reported R squared in the earlier version as it is helpful to see the explanatory power of the model. In the revised version, we report R squared values at the bottom part of the Table 4. The R squared increases from 2 to 7.9 %, indicating a moderate explanatory power, that is in line with R in other experiments where the focus is on the significance of the experimental explanatory variables more than on a complete model to explain the dependent variable.**

**It is true that subjects received information on whether they were winners (or losers) at the same time than they were informed on whether their group had overcome the effort of the rival group, and thus these two variables are correlated. However, the dummy variables that we use correspond to different events and are thus orthogonal by construction (the information they received determined uniquely if their group had been in the previous round winner by chance / winner deserved / loser by chance / loser deserved). We clarify now it in the text, when we introduce our specification 2, as follows (new text in bold):**

**“In specification 2 we add the effect of losing or winning deservedly or by chance in the previous round, deserved loser being the baseline case. Note that subjects received information on whether their group had overcome the effort of the rival and had win or lost, and therefore information on winning and losing and having done so deservedly or by chance was correlated. However, that information uniquely determined if the group had been a winner or a loser by chance or deservedly, and therefore the included dummy variables are orthogonal by construction.”**

3. Both in the main text and in the S2 Appendix it appears that the threshold for statistical significance is 0.1. Is that correct? This is not a big issue for me, yet it is common to consider the threshold at 5% and thus the results of the Wilcoxon signed-rank test (p-value=0.085) would not be significant.

**Throughout the main text and supplementary material we contemplate the results which are statistically significant at 10, 5 and 1 % of significance level as it can be found in Table 4 and 5, however, we completely agree with referee’s suggestion regarding the commonly considered significance level of 5%. Thus, we rephrased the sentence in S2 Appendix below Figure 2 as follows (new text in bold):**

**“A statistically significant lower effort at 10% in the last 5 rounds is found relative to the first 5 rounds (Wilcoxon signed-rank test, p-value= 0.085).”**

Overall, I believe the results presented in the experiment are interesting, yet they need to be stated in a more clear and transparent manner. In particular, Table 2 could benefit from a **visual representation** for at least some of the results, using, for instance, a bar plot.

**We appreciate referee’s suggestion to make use of visual representation to enhance the comprehension of our findings, a point also shared by the other referee. For the individuals from category “overall” in Table 2, we have elaborated Figure 1 in which we illustrate the share of individuals reacting to their own contribution in previous round relative to the group average. We introduced the Figure 1 at the beginning of section 3 called “Findings”.**

**We consider that this simplifies the message, and thus allows an easier understanding of our results. We comment on this as follows at the beginning of the Results section, as follows:**

**“Figure 1 indicates the share of participants who decided to increase or decrease their contribution depending on their behavior with respect to the group in the previous round. It shows that participants increase their contribution more frequently after having contributed less than the group average in the previous round, and that they decrease their contribution more frequently after having contributed more than the group average in the previous round. This suggests that there is conditional contribution in the group contest, the phenomenon being more emphatic upon having contributed less than the group average in the previous round.”**

**Minor comments**

The topic of conditional behavior in groups is very interesting. However, the group contest presented in this manuscript has some similarities with threshold public good games, in the sense that it encompasses both cooperative behavior towards the group as well as competition. Particularly, the study presented in [3] shows experimentally how conditional behaviour emerges in this type of games. How does your study compare to it?

**We thank the referee for pointing out the similarities with this work. As we do, it also identifies conditional cooperators in an environment related but different from the Public Good Game (group contest in our case, collective risk dilemma in the case of Fernandez Domingos et. al., 2020). We introduce it in the current version of the paper as follows:**

**“In an overview, Thoni and Volk (2018) report that the findings are quite stable across studies, about 61\% of participants being classified as conditional cooperators (followed by about 20\% of free-riders). This behavior has been described in other related environments, for instance, in collective-risk social dilemmas, where a group must achieve a given threshold through common contributions to avoid a general loss (i.e., as a climate change environment), see Fernandez Domingos et al. (2020)**.**”**

**There are also some minor typos and format issues in the paper:**

**1.** Line 187: There is a space missing after the point: “[…] to move towards the group average.As a robustness check […]”

**Corrected.**

**2.** Line 220: There is something wrong in this phrase: “[…] reduces contribution [change up to 13 pp], relative […]”

**We rephrased that part, as follows:**

**“In specification 2, we find that being in a group that loses by chance is the only group outcome that has a significant effect. The estimated coefficient indicates that it reduces contribution change in 13 pp, relative to deserved loser groups, as conjectured (see Table 1).”**

**3.** The **font size** in the tables should be bigger to increase readability.

**Thanks for pointing out this issue. We increased the font size.**

**4.** The table captions should contain more explanations about what is shown. In particular in Table 3, the **type** of regression performed should be indicated. Authors should also indicate the **R^2** value in case this was a linear regression.

**We acknowledge that the table captions were not informative enough in the previous version. We added R squared values to Table 4 (Table 3 in the previous version) and indicate that it is random effects linear panel regression model both at the bottom of the table and also in the caption.**

References

[1] <https://statisticsbyjim.com/regression/interpret-coefficients-p-values-regression/>
[2] <https://statisticsbyjim.com/regression/low-r-squared-regression/>
[1] Domingos, E. F., Grujić, J., Burguillo, J. C., Kirchsteiger, G., Santos, F. C., & Lenaerts, T. (2020). Timing uncertainty in collective risk dilemmas encourages group reciprocation and polarization. arXiv preprint arXiv:2003.07317.

**Reviewer #2:**

In this manuscript, the authors explore the determinants of conditional cooperative behavior in group contests. The results show that conditional cooperative behavior can appear in the successful group and those who contribute more than the rivals. Furthermore, this result is also verified by the random effect linear regression model. This paper has a good motivation.

However, there are some remaining issues with the manuscript, requiring some answers.

**We are grateful for the careful reading of our study and for pointing out ways to improve it.**

1) The setting of the first round of the experiment was unclear.

**The difference between the first round and the next rounds was that at the beginning of the first round there were no information about the previous round. We have clarified this point by explaining this difference, see last phrase below:**

**“At the end of each round, each participant received information i) on the amount of competition tokens that she bought; ii) on the total amount of competition tokens of the group; iii) on the total amount of competition tokens of the rival group; iv) on whether the group the participant belongs won the contest; v) on the individual payoff in the round. Note, therefore, that the first round was informatively different from the rest of rounds, because subjects did not have the additional information on the results of the previous round.”**

2) Previous experimental studies have shown that the number of rounds has an effect on the evolutionary results. The authors should discuss why the number of experimental rounds is set as 20 instead of the case that players are not informed the concrete game round.

**We wanted to keep our study similar to previous experimental contest games in order to test conditional cooperation in a well stablished setup, so we follow Abbink et al. (2010) in order to choose the main parameters of experiment. We note that other studies also use a fixed number of rounds, for instance**

**Chowdhury, S. M., Jeon, J. Y., & Ramalingam, A. (2016). Identity and group conflict. European Economic Review, 90, 107-121.**

**It is true that with this setup we are not avoiding the last-round effect, so we make it clear when describing the experiment, as follows (new text in bold):**

**“For the group contest, 14 groups of four were formed randomly and anonymously. Although making uncertain the number of rounds would avoid the last-round effect, to have a setup comparable with previous experiments on group contests, we follow Abbink et al. (2010), so groups were fixed for the 20 rounds of the group contest and the rival group remained the same as well.”**

3) Does t-1 mean previous round? t-1 is used in many places in the article, but I don't understand it. Does it refer to the previous round or all previous rounds? Concretely, see page 5, line 160. In addition, t should be italicized.

**We have clarified it when introducing Table 2. In particular, we modify the text slightly in two places to make clear that t-1 refers to the previous round:**

**“In Table 2 we show how participants reacted to when they contributed less / equal / more than the average in the previous round (that we denote as round *t-1*), conditional on the result of the group contest.**

**…**

**Table 2 depicts all these cases, where we provide the percentage of subjects that choose to increase or decrease their contribution in period t given their behavior with respect to their group in *t-1*.”**

**We have italicized the *t*-s in all the paper.**

4) The probability of players using C and D at the beginning of the public goods game should be explained.

**We are not completely sure about this comment of the referee. In the public goods game that our subjects played in phase 3 of the second part of the experiment, they had to choose a contribution in [0,1000]. In the bomb risk elicitation task that we used in order to elicit risk attitudes, we use different scenarios to explain how the task works, including scenario C and D. Since both situations are presented correlatively in the instructions (public goods game being Phase 3 and risk elicitation task being Phase 4), it could be misleading. In order to prevent that, we introduce now briefly the Instructions in the Appendix, in order to make it easier to separate each different part. The new text immediately before the Instructions is as follows:**

**“The language of the instruction was Spanish as we ran the experiment in Spain. Here we present the English version of the instructions. The instructions clarified the different tasks related to the Group Contest (Phase 1), Social Value Orientation (Phase 2), Public Goods game (Phase 3), Bomb Risk elicitation Task (Phase 4) and Competitiveness (Phase 5), as well as the Questionnaire.”**

5) There are some improper spaces in the typesetting of manuscripts, which leads to the destruction of the coherence of sentences and affects the reading. In addition, some expressions are not standardized.

**We have read carefully several times the paper and attempted to improve its readability. We removed improper spaces. We also tried to make the expressions more standardized (e.g. we use now only the term of round instead of using both rounds and periods in the previous version. We believe that the new version has a better quality, but we are ready to make it better if the referee finds it necessary (e.g. sending to a native speaker).**

6) Using some histogram or line chart can describe the change of contribution level in the game process more clearly.

**We agree with the referee that a nice graph is worth entire paragraphs. In the new version we add Figure 1 that depicts the reaction of the subjects to their contributions in the previous rounds, suggesting the presence of conditional cooperation. Moreover, in S2 Appendix we have now a figure showing average individual contribution per period with 95% confidence intervals. This figure indicates a slight downward trend in contributions that affects our analysis (e.g. the need to include a Round variable in the regression analysis).**

7) It is better to provide 95% confidence interval when analyzing linear regression model.

**As suggested by the referee regarding the confidence intervals provision as a valuable information, we present them in Table 7, in the S3 Appendix.**
