# Supplementary material for: Conditional cooperation in group contests
Source: PLoS One. 2020 Dec 23;15(12):e0244152. doi: 10.1371/journal.pone.0244152 (PMC7757887; doi:10.1371/journal.pone.0244152)
Supplement: Attachment — Submitted filename: Reports_with answers.docx [file pone.0244152.s006.docx]

PONE-D-20-25168R1
Conditional cooperation in group contests
PLOS ONE

Dear Dr. Kiss,

Thank you for submitting your manuscript to PLOS ONE. After careful consideration, we feel that it has merit but does not fully meet PLOS ONE’s publication criteria as it currently stands. Therefore, we invite you to submit a revised version of the manuscript that addresses the points raised during the review process.


Please submit your revised manuscript by Dec 25 2020 11:59PM. If you will need more time than this to complete your revisions, please reply to this message or contact the journal office at plosone@plos.org. When you're ready to submit your revision, log on to https://www.editorialmanager.com/pone/ and select the 'Submissions Needing Revision' folder to locate your manuscript file.

Please include the following items when submitting your revised manuscript:

- A rebuttal letter that responds to each point raised by the academic editor and reviewer(s). You should upload this letter as a separate file labeled 'Response to Reviewers'.
- A marked-up copy of your manuscript that highlights changes made to the original version. You should upload this as a separate file labeled 'Revised Manuscript with Track Changes'.
- An unmarked version of your revised paper without tracked changes. You should upload this as a separate file labeled 'Manuscript'.

If you would like to make changes to your financial disclosure, please include your updated statement in your cover letter. Guidelines for resubmitting your figure files are available below the reviewer comments at the end of this letter.

If applicable, we recommend that you deposit your laboratory protocols in protocols.io to enhance the reproducibility of your results. Protocols.io assigns your protocol its own identifier (DOI) so that it can be cited independently in the future. For instructions see: <http://journals.plos.org/plosone/s/submission-guidelines#loc-laboratory-protocols>

We look forward to receiving your revised manuscript.

Kind regards,

The Anh Han, Ph.D.
Academic Editor
PLOS ONE


Additional Editor Comments (if provided):

Both reviewers have agreed that revised version has been improved, but their previous comments have only been partially addressed. There are still a few aspects remained to be dealt with as suggested by the reviewers, which the authors should carefully consider in their revision.


[Note: HTML markup is below. Please do not edit.]

Reviewers' comments:

Reviewer's Responses to Questions

**Comments to the Author**

1. If the authors have adequately addressed your comments raised in a previous round of review and you feel that this manuscript is now acceptable for publication, you may indicate that here to bypass the “Comments to the Author” section, enter your conflict of interest statement in the “Confidential to Editor” section, and submit your "Accept" recommendation.

Reviewer #1: All comments have been addressed

Reviewer #2: (No Response)

2. Is the manuscript technically sound, and do the data support the conclusions?

The manuscript must describe a technically sound piece of scientific research with data that supports the conclusions. Experiments must have been conducted rigorously, with appropriate controls, replication, and sample sizes. The conclusions must be drawn appropriately based on the data presented.

Reviewer #1: Yes

Reviewer #2: Yes

3. Has the statistical analysis been performed appropriately and rigorously?

Reviewer #1: Yes

Reviewer #2: Yes

4. Have the authors made all data underlying the findings in their manuscript fully available?

The [PLOS Data policy](http://www.plosone.org/static/policies.action#sharing) requires authors to make all data underlying the findings described in their manuscript fully available without restriction, with rare exception (please refer to the Data Availability Statement in the manuscript PDF file). The data should be provided as part of the manuscript or its supporting information, or deposited to a public repository. For example, in addition to summary statistics, the data points behind means, medians and variance measures should be available. If there are restrictions on publicly sharing data—e.g. participant privacy or use of data from a third party—those must be specified.

Reviewer #1: No

Reviewer #2: Yes

5. Is the manuscript presented in an intelligible fashion and written in standard English?

PLOS ONE does not copyedit accepted manuscripts, so the language in submitted articles must be clear, correct, and unambiguous. Any typographical or grammatical errors should be corrected at revision, so please note any specific errors here.

Reviewer #1: Yes

Reviewer #2: Yes

6. Review Comments to the Author

Please use the space provided to explain your answers to the questions above. You may also include additional comments for the author, including concerns about dual publication, research ethics, or publication ethics. (Please upload your review as an attachment if it exceeds 20,000 characters)

Reviewer #1: The authors have addressed all my comments. And the new figure added largely help the understanding of the main message of the article. In particular, now, in Figure 1 it is clear that there is a conditional effect with most participants increasing/decreasing their contributions if they previously contributed less / more than the group’s average in the previous round. Thus I recommend that this paper is accepted. Yet, some parts of the paper are still not clear and I would prefer that the authors review these parts before the paper is published:

Figure 1: It is not clear if these are overall results, or if they take into account only the conditions in which players won/lost. This is particularly important since later in Table 2 you do condition the results on win/loss. It would be good to clarify both in the text and the Figure’s Caption that this are results averaged over all data points. Moreover, there should be more information about the sample used in this figure (how many data points are used for each bar – remember you are using percentages).

***In the new version we made clear that Fig 1 refers to the overall results. We also explained that actually it shows the data in the rows „overall” of the top and bottom panels of Table 2. The new versions reads as „Fig 1 indicates the share of participants who decided to increase or decrease their 159 contribution depending on their behavior with respect to the group in the previous 160 round , averaged over all decisions. (In Fig 1 we consider all the decisions unconditionally, while Table 2 161 also indicates the corresponding numbers conditional on winning / losing and the 162 nature of winning and losing. Hence, Fig 1 depicts the row overall of the top and 163 bottom panels in Table 2.)”***

***The new version makes clear both in the text and the caption that Fig 1 presents results averaged over all data points. We have included in the caption of Fig 1 the number of observations for both bar diagrams, so given the shares depicted in Fig 1 the reader knows the number of data points used for each bar.***

Table 3: It appears that the magnitude of decrease in all cases is higher than that of increase. Which indicates that in all cases participants are more careful when increasing their contributions. Moreover, the magnitude of increase is bigger when players already contributed more than the average in the previous round, why does this happen? Is it possible that not only the previous round matters in this case?

***The referee raises very interesting issues with her / his observations. The fact that decreases are larger than increases is partly explained by the fact that there is a downward trend in the level of contributions that implies that overall decreases are larger than increases. We have no good explanation on why the size of the increase is larger when players contributed more than the average in the previous round. This is a finding that we did not expect and we do not have a clear explanation for it. Following the later suggestion of the referee, we explicitely include this as a limitation in the new „Conclusion and Discussion” section that replaces the previous „Conclusion”. We find these observations intriguing and worth calling the attention of the readers to them, as follows:***

***„While these findings reveal the role of conditonal cooperation in group contests, it is important to consider some limitations. We lack an explanation for some of the asymmetric behaviors observed in the experiment. In particular, contributions are larger when contribution in the previous round was already larger than the group average relative to the case when contribution in the previous round was smaller than the group average.”.***

Also, it seems surprising that the majority of participants that contributed as much as the group average, decrease their contributions with the biggest change in all 3 conditions (>, =, <), and this happens independently on whether the group lost or won the contest. Is there some explanation to this?

**We thank the referee for raising this point, because we have understood that the previous version of the Table was misleading. The reason for this fact was that due to a coding error we had included the actions chosen in the first period (t=1), which accounted for 56 (all first period decisions) out of the 63 observations of agents who had contributed excatly the same than the average in the previous round. In the new version of the Table we exclude these observations. In the 7 remaining cases of contributing exactly the same than the average in t-1, the results are not different from the other cases. It is consistent with the robustness check that we included in the Appendix, where we had the same table but defining the same contribution as contributions which differ from the average in less than +/-20%. Since we have only 7 cases we did not include in the table the conditional percentages (loser / winner etc.).**

**We checked carefully our codes to make sure that there is no other error in them. Fortunately, this was the only one so the previous findings and their interpretations hold.**

Table 4: The R^2 values are quite small. I would like to see at the end of the paper some comment about the limitations of the study, both in this regard and in general. Overall, I do miss this critical overview of the work that help readers understand the limitations of the study and thus apply the conclusions with care.

**We changed the Conclusion section to Conclusion and Discussion, thus the new version contains a discussion of our findings after the conclusion. We agree with the reviewer that such a discussion was sorely missing from the previous version. Our main point with this paper was to show that conditional cooperation as a phenomenon applies also to group contests. We believe that we make a convincing point that it is the case. However, we also indicate that it does not apply in all conditions (e.g. deserved loser) and that we observe also surprising things (for instance the ones pointed out by the reviewer). Hence, in the discussion we highlight these points. Moreover, we call the attention to two further limitations. On the one hand, we analyze data from just one experiment so to obtain more robust findings data from other group contest experiments should be analyzed as well. On the other hand, and relatedly to the previous point, group contests come in many format (see for instance the nice survey by Sheremeta, R. M. (2018). Behavior in group contests: A review of experimental research. Journal of Economic Surveys, 32(3), 683-704.) and we do not know to what degree our findings are format-depending, due to some specific element of the experimental design. Overall, a meta-analytic study would help to shed light on these issues. We believe that such an exercise is beyond the scope of this paper, but we plan to do it in the future.**

Figure 2 should be Figure S2. Moreover, it appears that there are no significant individual changes in contributions per round.

Finally, while in the text you name “Figure 1” or “Figure 2”, you label figures as “Fig. 1” and “Fig. 2”, this should be corrected. Moreover, in the text that you have already corrected you write: “In S2 Appendix we present evidence…”. I believe you mean “In Figure S2 of the Appendix, we present evidence…”. It would be good if the authors reread the paper once more to eliminate these typos.

**Typos have been corrected.**

Reviewer #2: The authors have addressed most of my comments, but a few issues with the paper's clarity still remain for me and should be seen to before publication.

In page 1, line 6, "[8] report that..." should be corrected as "[8] reports that...";
In page 2, line26, "[30] find that..." should be corrected as "[30] find that...";
In page 2, line 27, "[7] report that..." should be corrected as "[7] reports that..."

**This typos have been corrected.**

The authors should examine the manuscript carefully to avoid these grammatical problems. In addition, there are also some English writing problems in "the experiment" section, especially tenses, which need to be carefully proofread.

**We thank the referee for pointing out these problems and inconsistencies in the text. They have been corrected.**

In dealing with the problem of promoting the evolution of cooperative behavior, there are many related papers worth reviewing, such as Cost effective external interference for promoting the evolution of cooperation, 8:15997 (2018)，Evolutionary dynamics of cooperation in a population with probabilistic corrupt enforcers and violators, 29(11): 2127­-2149 (2019).

**We thank the referee for her/his suggestions, which we have included in order to enrich the literature on evolution of cooperative behavior.**

7. PLOS authors have the option to publish the peer review history of their article ([what does this mean?](https://journals.plos.org/plosone/s/editorial-and-peer-review-process#loc-peer-review-history)). If published, this will include your full peer review and any attached files.


If you choose “no”, your identity will remain anonymous but your review may still be made public.


**Do you want your identity to be public for this peer review?** For information about this choice, including consent withdrawal, please see our [Privacy Policy](https://www.plos.org/privacy-policy).

Reviewer #1: No

Reviewer #2: No

[NOTE: If reviewer comments were submitted as an attachment file, they will be attached to this email and accessible via the submission site. Please log into your account, locate the manuscript record, and check for the action link "View Attachments". If this link does not appear, there are no attachment files.]

While revising your submission, please upload your figure files to the Preflight Analysis and Conversion Engine (PACE) digital diagnostic tool, <https://pacev2.apexcovantage.com/>. PACE helps ensure that figures meet PLOS requirements. To use PACE, you must first register as a user. Registration is free. Then, login and navigate to the UPLOAD tab, where you will find detailed instructions on how to use the tool. If you encounter any issues or have any questions when using PACE, please email PLOS at [figures@plos.org](mailto:figures@plos.org). Please note that Supporting Information files do not need this step.
